# Serum lipidomic alterations associated with post-stroke cognitive impairment: an exploratory prospective study

**DOI:** 10.3389/fneur.2026.1882198

**Published:** 2026-07-29

**Authors:** Xinyu Zhang, Xiangwen Hao, Jingqi Chen, Yanyang Li, Bianying Feng, Ailipinai Yasen, Zhen Dan, Xiaoyan Zheng, Anren Zhang, Qiuhong Man

**Affiliations:** 1 School of Health Preservation and Rehabilitation, Chengdu University of Traditional Chinese Medicine, Chengdu, China; 2 Department of Clinical Laboratory, Shanghai Fourth People's Hospital, School of Medicine, Tongji University, Shanghai, China; 3 Rehabilitation Medicine Center, The First Affiliated Hospital of Nanjing Medical University, Nanjing, China; 4 Department of Rehabilitation Medicine, Shanghai Fourth People’s Hospital Affiliated to Tongji University, Shanghai, China

**Keywords:** biomarkers, cognitive impairment, lipidomics, PSCI, stroke

## Abstract

**Background:**

Post-stroke cognitive impairment (PSCI) is a common complication of stroke that adversely affects long-term functional outcomes and quality of life among survivors. However, reliable biomarkers for early risk prediction remain limited. Dysregulation of lipid metabolism has been implicated in ischemic stroke and may contribute to PSCI development.

**Objective:**

This study investigated early serum lipidomic alterations associated with PSCI in patients with acute ischemic stroke and evaluated whether integrating lipidomic features with clinical indicators improves predictive performance.

**Methods:**

In this prospective cohort study, 123 patients with acute ischemic stroke were enrolled. Serum samples were obtained within 24 h of stroke onset, and untargeted lipidomic profiling was performed using ultra-high-performance liquid chromatography–high-resolution mass spectrometry. Cognitive function was assessed at 3 months using the Telephone Montreal Cognitive Assessment (T-MoCA). Patients were classified into PSCI and post-stroke cognitively normal (PSCN) groups. Candidate lipids were identified through univariate analysis, multivariable logistic regression, pathway enrichment analysis, and least absolute shrinkage and selection operator (LASSO) regression. Clinical, lipidomic, and combined prediction models were developed and internally validated.

**Results:**

Among the 123 patients, 62 developed PSCI and 61 remained cognitively normal. A total of 1,584 lipid annotations meeting MSI Level 2 criteria were retained for subsequent analyses. Although 87 lipids differed nominally between the PSCI and PSCN groups, none remained significant after false discovery rate correction. After adjustment for clinical confounders, 65 lipids remained nominally associated with PSCI. Pathway enrichment analysis implicated glycerophospholipid metabolism and choline-related phospholipid metabolism. LASSO regression identified LPC 22:6/0:0 as a candidate lipidomic feature. In the internal validation set, the clinical, lipidomic, and combined models yielded areas under the curve (AUCs) of 0.746, 0.623, and 0.763, respectively. The combined model demonstrated favorable discrimination and calibration, as well as potential clinical net benefit.

**Conclusion:**

These findings suggest early serum lipid metabolic alterations in patients who developed PSCI. LPC 22:6/0:0 may be associated with post-stroke cognitive outcomes, and a model integrating clinical and lipidomic features may support early PSCI risk stratification.

## 1 Introduction

Post-stroke cognitive impairment (PSCI) is a common and clinically important complication after stroke (1, 2) that adversely affects quality of life, rehabilitation outcomes, and social reintegration in stroke survivors and is associated with increased risks of dementia and mortality. Previous studies have shown that the prevalence of PSCI varies according to population characteristics, follow-up duration, and diagnostic criteria, with reported estimates ranging from 20 to 75% (3–5). The onset of PSCI is often insidious and easily overlooked in the early stage. Patients may exhibit impairments across multiple cognitive domains, including attention, executive function, memory, language, and visuospatial ability. In addition, some patients may develop deficits in social cognition (6–9). Currently, the early clinical identification of PSCI mainly relies on neuropsychological assessments and neuroimaging examinations, such as the Mini-Mental State Examination (MMSE), the Montreal Cognitive Assessment (MoCA), cranial computed tomography (CT), and structural magnetic resonance imaging (MRI) (10–12). However, these approaches have several limitations: neuropsychological scales are influenced by educational level, language ability, emotional status, and examiner-related factors, whereas neuroimaging examinations are limited by high cost, time requirements, and restricted accessibility. Specifically, CT involves exposure to ionizing radiation, whereas MRI is radiation-free but requires a longer acquisition time and may not be suitable for some patients. Therefore, simple, objective, and readily accessible blood-based biomarkers are urgently needed for the early identification of PSCI in clinical practice.

As essential biomolecules, lipids not only contribute to cell membrane architecture and energy metabolism but also play key roles in maintaining cellular homeostasis and mediating signal transduction (13). In the central nervous system, lipids are extensively involved in myelination, the maintenance of synaptic function, and the regulation of neuronal membrane homeostasis; therefore, dysregulated lipid metabolism is closely associated with a wide range of neurological disorders (14). In recent years, lipidomics studies have identified disease-specific alterations in lipid profiles in Alzheimer’s disease, Parkinson’s disease, and stroke, which may contribute to disease classification, mechanistic investigation, and risk assessment (15–22). Collectively, these findings suggest that lipidomics has substantial potential for biomarker discovery and pathophysiological characterization in neurological diseases.

Accumulating evidence suggests that the occurrence of ischemic stroke is associated with dysregulated lipid metabolism, which may also be closely involved in the development of PSCI (23). Potential mechanisms include the promotion of atherosclerosis and cerebral small vessel disease, aggravation of cerebral hypoperfusion and white matter injury, disruption of blood–brain barrier integrity, and induction of neuroinflammation, apoptosis, and neurovascular unit dysfunction (24–26). Therefore, abnormal lipid metabolism may act both as an upstream driver of ischemic injury progression and as an important pathological contributor to post-stroke cognitive decline. However, systematic lipidomic investigations of PSCI, particularly those spanning the acute to recovery phases after stroke, remain scarce. Most existing studies have focused on a limited number of candidate lipids, without comprehensively characterizing the dynamic evolution of lipid profiles or providing longitudinal evidence from prospective cohorts linking lipid alterations to long-term cognitive outcomes.

Accordingly, this prospective cohort study enrolled patients with acute ischemic stroke and applied mass spectrometry-based untargeted lipidomics to characterize serum lipid profiles within 24 h of symptom onset, followed by cognitive assessment at 3 months after stroke. According to the 3-month T-MoCA scores, patients were classified into the PSCI group and the PSCN group, and acute-phase lipid metabolic characteristics were systematically compared between the two groups. Key lipid molecules and metabolic pathways associated with post-stroke cognitive outcomes were further identified. Clinical indicators and lipidomic features were further integrated to develop and validate an early prediction model for PSCI, and its predictive performance, calibration, and clinical utility were evaluated. This study aimed to elucidate acute-phase lipid metabolic abnormalities associated with the development of PSCI and to provide evidence for early identification, risk stratification, and exploration of potential intervention targets in patients at high risk for post-stroke cognitive impairment.

## 2 Materials and methods

### 2.1 Study population and data collection

This study was conducted in accordance with the Declaration of Helsinki and was approved by the Ethics Committee of Shanghai Fourth People’s Hospital (Protocol No. 2024056–001). The study was registered with the Chinese Clinical Trial Registry on March 29, 2024 (ChiCTR2400082449). Written informed consent was obtained from all participants or their legally authorized representatives.

Consecutive patients with acute ischemic stroke admitted to Shanghai Fourth People’s Hospital between January 2024 and February 2025 were enrolled. Pretreatment serum samples for untargeted lipidomic analysis were obtained from all enrolled patients within 24 h of symptom onset. Demographic characteristics, medical history, medication history, clinical data, and laboratory findings were collected at the same time point. Demographic variables included age and sex. Medical history included smoking history, alcohol consumption, surgical history, history of stroke, hypertension, diabetes mellitus, coronary heart disease, dyslipidemia, and atrial fibrillation. Medication history included the use of antiplatelet agents, anticoagulants, antihypertensive agents, and glucose-lowering medications. Clinical data included the National Institutes of Health Stroke Scale (NIHSS) score at admission, Glasgow Coma Scale (GCS) score, intravenous thrombolysis status, and Trial of Org 10,172 in Acute Stroke Treatment (TOAST) etiologic classification. Laboratory indicators included small dense low-density lipoprotein cholesterol (sdLDL-C), apolipoprotein B, apolipoprotein E, apolipoprotein A-I, homocysteine, folate, vitamin B12, glycated hemoglobin, and glucose. Stroke severity was assessed by trained clinicians using the NIHSS. The GCS score ranges from 3 to 15, with higher scores indicating a higher level of consciousness. Stroke etiology was classified according to the TOAST criteria.

### 2.2 Inclusion and exclusion criteria

The inclusion criteria were as follows: (1) age ≥18 years; (2) ischemic stroke confirmed by brain computed tomography (CT) or magnetic resonance imaging (MRI); (3) collection of serum samples within 24 h of symptom onset; (4) ability of the patient or a legally authorized representative to provide written informed consent; and (5) willingness to complete 3-month cognitive follow-up after stroke.

The exclusion criteria were as follows: (1) pre-existing cognitive impairment, a history of psychiatric disorders, brain tumor, traumatic brain injury, or other conditions that could affect cognitive function; (2) inability to complete the cognitive assessment because of severe aphasia, hearing impairment, impaired consciousness, severe communication difficulties, or writing difficulties; (3) use of cholesterol-lowering or other lipid-lowering medications within the preceding 6 months; (4) refusal to complete the cognitive assessment or follow-up evaluations; and (5) pregnancy or lactation.

A total of 206 patients with acute ischemic stroke were initially screened. Of these, 83 were excluded for the following reasons: pre-existing cognitive impairment (*n* = 18), severe aphasia (*n* = 22), history of psychiatric disorders (*n* = 9), pre-stroke lipid-lowering medication use (*n* = 7), and refusal to participate in follow-up (*n* = 27). Ultimately, 123 patients were included in the final analysis, all of whom completed the 3-month cognitive assessment after stroke.

### 2.3 Cognitive assessment and outcome definition

Cognitive function was assessed 3 months after stroke by standardized, trained evaluators who were blinded to patients’ clinical data and lipidomic results. The T-MoCA was used for follow-up assessment to reduce attrition associated with difficulty attending face-to-face visits (27). The T-MoCA has a total score of 22 points and assesses cognitive domains including attention, language, abstraction, delayed recall, and orientation (28). The primary outcome was PSCI. To avoid classification bias resulting from including acute-phase cognitive status in the outcome definition, PSCI in the primary analysis was defined solely according to the T-MoCA score 3 months after stroke. Patients with a T-MoCA score of <19 were classified as having PSCI, whereas those with a T-MoCA score of ≥19 were classified as PSCN (28–30).

### 2.4 Blood sample collection and lipid extraction

Venous blood samples (10 mL) were collected within 24 h of symptom onset by standard venipuncture and placed in serum separation tubes. Samples were allowed to stand at room temperature for 30 min and then centrifuged at 4,000 × g for 10 min at 4 °C. After serum separation, 500-μL aliquots were prepared and immediately stored at −80 °C until lipidomic analysis.

Before analysis, samples were thawed at 4 °C. A 50-μL serum aliquot was transferred, and 150 μL of extraction reagent was added. The mixture was mixed on a vortex shaker at 1,500 rpm for 20 min. Subsequently, the 96-well plate was centrifuged at 2,200 × g for 10 min at 4 °C. After centrifugation, the supernatant was collected and filtered through a protein precipitation plate. The filtrate was analyzed on a C18 chromatographic column in both positive and negative ion modes.

### 2.5 Ultra-high-performance liquid chromatography–mass spectrometry analysis

Untargeted lipidomic profiling was performed using a Thermo Scientific Vanquish ultra-high-performance liquid chromatography system coupled to a Q Exactive quadrupole-Orbitrap high-resolution mass spectrometer. Chromatographic separation was achieved on an ACQUITY BEH C18 column (2.1 × 100 mm, 1.7 μm) at a column temperature of 40 °C and a flow rate of 0.3 mL/min.

The same mobile phase system was used in both positive and negative ion modes. Mobile phase A consisted of 60% acetonitrile in water containing 2 mM ammonium formate and 0.1% formic acid. Mobile phase B consisted of isopropanol/acetonitrile (90:10, v/v) containing 2 mM ammonium formate and 0.1% formic acid. The gradient elution program was as follows: mobile phase B was initially set at 30%, linearly increased to 100% over 20 min, and then maintained at 100% for 5 min.

Mass spectrometric detection was performed using a heated electrospray ionization source operated in both positive and negative ion modes. Full-scan MS spectra were acquired over an m/z range of 200–2,000 at a resolution of 70,000 full width at half maximum (FWHM). The automatic gain control (AGC) target was set to 1 × 10^6, and the maximum injection time was 100 ms. Tandem mass spectrometry was performed in data-dependent acquisition (DDA) mode, in which the 10 most abundant precursor ions were selected for fragmentation. The MS/MS resolution was 17,500 FWHM, the AGC target was 3 × 10^6, the maximum injection time was 50 ms, and the dynamic exclusion time was 4 s. In negative ion mode, stepped normalized collision energies of 20 and 30% were used.

### 2.6 Lipid annotation, annotation confidence, and relative quantification

Raw mass spectrometry data files (.raw) were converted to Analysis Base File (.abf) format using the Reifycs ABF Converter. The converted files were subsequently imported into MS-DIAL version 4.80 (RIKEN Center for Sustainable Resource Science) for untargeted lipidomics data processing, lipid annotation, and peak area–based relative quantification.

The data processing workflow comprised three main steps: (1) peak picking and chromatographic alignment, (2) isotope and adduct deconvolution, and (3) lipid annotation. Unless otherwise specified, the default MS-DIAL parameters optimized for high-resolution Orbitrap data were used. For peak detection, the mass slice width was set to 0.05 Da, and a minimum peak height of 100,000 amplitude was applied to filter low-intensity noise. Chromatographic alignment across samples was performed using a retention time tolerance of 0.1 min and an MS1 tolerance of 0.1 Da.

For lipid annotation, the identification score cutoff was set to 80%. Accurate mass tolerances were set to 0.01 Da for MS1 and 0.025 Da for MS/MS, and the retention time tolerance was set to 0.1 min. MS-DIAL uses a hybrid scoring system that integrates accurate mass, isotopic distribution, and MS/MS spectral matching. In this study, lipid annotation was primarily driven by MS/MS spectral matching against the LipidBlast MSP library, supplemented by lipid-class-specific fragmentation rules implemented in MS-DIAL/MS2Dec. Common adduct ions were considered according to ionization mode, including [M + H]^+^, [M + NH₄]^+^, and [M + Na]^+^ in positive ion mode, and [M − H]^−^ and [M − H₂O − H]^−^ in negative ion mode.

Because no authentic chemical standards were injected in parallel for all detected lipid species in the current untargeted DDA workflow, the 1,584 reported lipid features were assigned as MSI Level 2 annotations rather than MSI Level 1 confirmed identifications. These annotations were supported by accurate mass, MS/MS spectral matching to the LipidBlast library with an MS-DIAL composite score >80%, and agreement with lipid-class-specific neutral loss and fragment ion rules. Therefore, the lipid species reported in this study should be interpreted as putative lipid annotations rather than unequivocally confirmed molecular structures. Mixed isotope-labeled internal standards were used primarily for monitoring the extraction process, signal stability, relative quantification correction, and quality control, rather than for compound-specific structural confirmation of all lipid species.

### 2.7 Quality control, data normalization, and batch effect assessment

Quality control (QC) samples were prepared by pooling equal aliquots from all study samples and were processed according to the same pretreatment protocol used for the study samples. QC samples were used to evaluate instrument performance, condition the chromatography–mass spectrometry system, and monitor the stability of the entire analytical process. Before sample acquisition, the injection order of study samples was randomized. QC samples were distributed evenly throughout the analytical sequence, with one QC sample injected after every six to eight study samples.

Analytical reproducibility was evaluated using pooled QC samples. For each lipid feature, the relative standard deviation (RSD) across QC injections was calculated. Lipid features with a QC RSD of ≤30% were retained for subsequent analysis, whereas those with a QC RSD of >30% were excluded before statistical analysis. To further assess data quality, lipid intensity distributions across samples, QC RSD distributions, and unsupervised principal component analysis (PCA) were evaluated. Sample intensity distributions were used to assess the comparability of overall lipid signals, whereas PCA was used to evaluate QC sample clustering, potential sample outliers, and possible batch effects.

To mitigate potential batch effects, data normalization and block correction were performed in addition to randomizing the injection order and inserting QC samples at regular intervals. Specifically, each lipid feature was corrected using the within-block median, and lipid intensities were proportionally normalized. After missing value processing, normalization, QC filtering, and block correction, the final lipid intensity matrix was generated for subsequent statistical analyses. Missing values were processed as follows: lipid features with a missing rate >30% were removed, and the remaining missing values were imputed using the k-nearest neighbors (KNN) method.

### 2.8 Statistical analysis

All statistical analyses were performed using R software. Continuous variables are presented as the mean ± standard deviation or median with interquartile range, depending on their distribution, whereas categorical variables are presented as frequencies and percentages. The normality of continuous variables was assessed using the Shapiro–Wilk test, histograms, and quantile–quantile plots. For between-group comparisons, normally distributed continuous variables were analyzed using the independent-samples t-test, whereas non-normally distributed continuous variables were analyzed using the Mann–Whitney U test. Categorical variables were analyzed using the χ^2^ test or Fisher’s exact test, as appropriate. All statistical tests were two-sided, and unless otherwise specified, *p* < 0.05 was considered nominally significant.

Lipidomic analyses were conducted using the lipid intensity matrix generated after peak detection, lipid annotation, missing value processing, normalization, quality-control filtering, and batch correction. PCA was used to evaluate the overall data structure, QC sample clustering, potential sample outliers, and distribution trends between the PSCI and PSCN groups. Partial least squares discriminant analysis (PLS-DA) was used solely as an exploratory visualization method to examine separation trends between the two groups. The robustness of the PLS-DA model was evaluated using cross-validation and 200 permutation tests; model parameters, including R^2^X, R^2^Y, and Q^2^, were reported. Given the risk of overfitting associated with PLS-DA in small-sample, high-dimensional omics datasets, differential lipid selection was not based on PLS-DA results.

Differences in lipid abundance between the PSCI and PSCN groups were evaluated using univariate statistical tests. Fold change (FC) was calculated as the ratio of lipid abundance in the PSCI group to that in the PSCN group and expressed as the log₂-transformed fold change (log₂FC). Because a large number of lipid species were detected, raw *p* values were adjusted for the false discovery rate (FDR) using the Benjamini–Hochberg method, and the FDR value for each lipid was reported. Lipid features with FDR < 0.05 were considered significant after correction for multiple testing. Because this was an exploratory prospective cohort study and both comparison groups consisted of patients with acute ischemic stroke, lipid differences between the PSCI and PSCN groups were expected to be more subtle than those between patients and healthy controls. Therefore, lipids with raw *p* < 0.05 were defined only as nominally significant exploratory candidate lipids for hypothesis-generating analyses and were not regarded as confirmed differential lipids or validated biomarkers.

To assess whether associations between candidate lipids and PSCI were independent of potential clinical confounders, multivariable logistic regression was performed. PSCI status was used as the dependent variable, and each nominally significant candidate lipid was entered separately as an independent variable in the model. The models were adjusted for variables that differed between groups or were clinically relevant, including diabetes mellitus, glucose-lowering medication use, apolipoprotein E, and homocysteine. Adjusted 95% confidence intervals (CIs) and adjusted *p* values were calculated. Lipids that remained nominally associated with PSCI after adjustment for clinical confounders were used for subsequent exploratory visualization, pathway enrichment analysis, and prediction model development. Volcano plots, heatmaps, and lipid class distribution plots were used to illustrate the overall characteristics of candidate lipids.

Lipid pathway enrichment analysis based on the Kyoto Encyclopedia of Genes and Genomes (KEGG) was performed using an in-house bioinformatics workflow. Human pathway annotation files and compound–pathway mapping information were obtained from the 2024 version of the KEGG database. For lipids or metabolites with HMDB annotations, the 2024 version of the Small Molecule Pathway Database (SMPDB) was used only as a supplementary annotation source. In the enrichment analysis, all lipids retained after quality-control filtering were used as the background set, and candidate lipids were used as the input set. Fisher’s exact test was used to assess the enrichment significance of each pathway, and the Benjamini–Hochberg method was applied for multiple-testing correction. Pathways with FDR < 0.05 were considered significantly enriched, whereas pathways with *p* < 0.05 but without FDR significance were described only as exploratory findings. The enrichment factor was defined as the proportion of candidate lipids in a given pathway relative to the total number of background lipids in that pathway. Enrichment results were visualized using bubble plots.

For prediction model development, the study cohort was randomly divided into a training set and an internal validation set at a 7:3 ratio, and baseline characteristics were compared between the two sets to evaluate their balance. All variable selection, penalty parameter tuning, and model fitting were performed exclusively in the training set; the internal validation set was not used for variable selection or model training. In the training set, least absolute shrinkage and selection operator (LASSO) logistic regression was applied separately to clinical variables and exploratory candidate lipidomic variables for feature selection. The optimal penalty parameter *λ* was determined by 10-fold cross-validation. Variables with nonzero regression coefficients were then included in multivariable logistic regression models to develop the clinical, lipidomic, and combined models. Model discrimination was evaluated using receiver operating characteristic (ROC) curves, the area under the curve (AUC) with 95% CIs, sensitivity, and specificity. Differences in AUCs between models were compared using the DeLong test. Model calibration and clinical utility were assessed using calibration curves, Brier scores, 1,000 bootstrap resamples for internal validation, and decision curve analysis.

## 3 Results

### 3.1 Baseline characteristics

**Figure 1:**
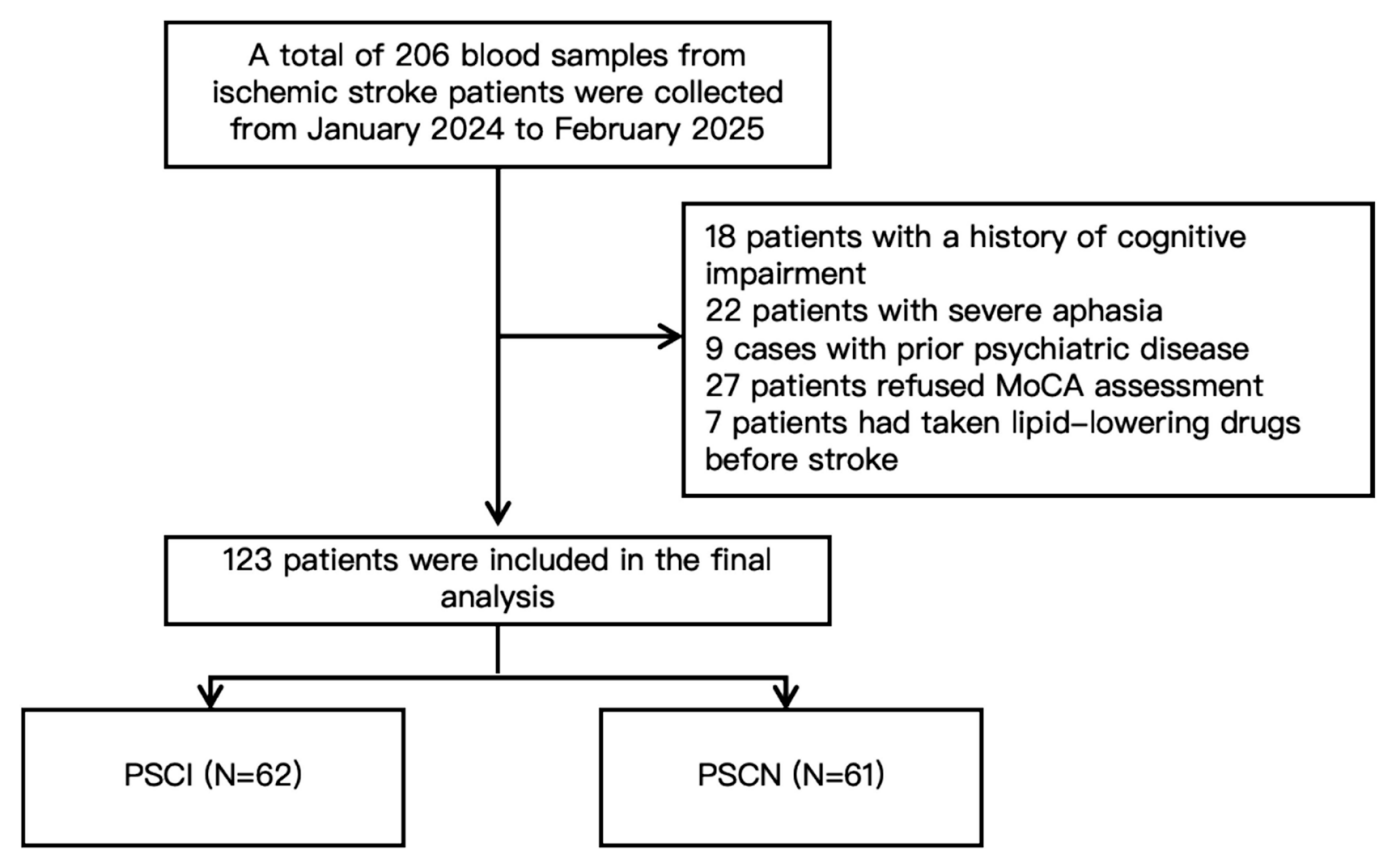
Flowchart of patient screening and enrollment.

A total of 123 patients with acute ischemic stroke were prospectively enrolled, all of whom completed the 3-month cognitive follow-up assessment. On the basis of the 3-month T-MoCA score alone, 62 patients were classified as having post-stroke cognitive impairment (PSCI group; 50.41%), and 61 were classified as post-stroke cognitively normal (PSCN group; 49.59%). The patient screening and enrollment process is presented in Figure 1. Baseline demographic, clinical, and laboratory characteristics are summarized in Table 1. No significant differences were observed between the PSCI and PSCN groups in age, sex, admission NIHSS score, admission GCS score, intravenous thrombolysis, TOAST etiologic classification, hypertension, coronary heart disease, atrial fibrillation, dyslipidemia, smoking, alcohol consumption, surgical history, antiplatelet therapy, anticoagulant therapy, or antihypertensive treatment (all *p* > 0.05). As expected based on the outcome definition, the 3-month T-MoCA score was significantly lower in the PSCI group than in the PSCN group (15.77 ± 2.70 vs. 20.02 ± 0.99, *p* < 0.001). Diabetes mellitus and glucose-lowering medication use were more frequent in the PSCN group than in the PSCI group (both *p* = 0.013). In contrast, apolipoprotein E levels were higher in the PSCI group than in the PSCN group (4.64 ± 1.63 vs. 3.94 ± 1.33 mg/dL, *p* = 0.011), as were homocysteine levels (20.57 ± 17.28 vs. 14.66 ± 5.75 μmol/L, *p* = 0.013). No significant differences were observed in small dense low-density lipoprotein cholesterol, apolipoprotein B, apolipoprotein A1, folate, vitamin B12, glycated hemoglobin, or glucose levels. Overall, most baseline variables were comparable between groups; however, diabetes mellitus, glucose-lowering medication use, apolipoprotein E, and homocysteine showed between-group differences and were therefore considered potential clinical confounders in subsequent adjusted analyses.

**Table 1:** Baseline characteristics of the study participants stratified by 3-month cognitive outcome.

Characteristic	PSCN (*n* = 61)	PSCI (*n* = 62)	*p* value
Clinical characteristics			
Age (years)	66.95 ± 10.38	69.40 ± 8.89	0.162
Male [*n* (%)]	43 (70.5%)	41 (66.1%)	0.699
Admission NIHSS score	2.80 ± 3.93	2.82 ± 3.98	0.978
TOAST classification (%)			0.721
Large artery atherosclerosis	45 (73.8)	47 (75.8)	
Small artery occlusion	0 (0.0)	0 (0.0)	
Cardioembolic	3 (4.9)	1 (1.6)	
Undetermined cause	13 (21.3)	14 (22.6)	
Admission GCS score	14.9 ± 0.6	14.8 ± 0.7	0.397
3-month T-MoCA score	20.02 ± 0.99	15.77 ± 2.70	<0.001
Smoking [*n* (%)]	9 (14.8)	9 (14.5)	>0.999
Alcohol use [*n* (%)]	6 (9.8)	6 (9.7)	>0.999
Hypertension [*n* (%)]	44 (72.1)	36 (58.1)	0.131
Diabetes mellitus [*n* (%)]	27 (44.3)	14 (22.6)	0.013
Coronary heart disease [*n* (%)]	6 (9.8)	4 (6.5)	0.530
Atrial fibrillation [*n* (%)]	3 (4.9)	1 (1.6)	0.365
History of surgery [*n* (%)]	10 (16.4)	8 (12.9)	0.619
Dyslipidemia [*n* (%)]	2 (3.3)	5 (8.1)	0.439
Thrombolysis [*n* (%)]	12 (19.7)	11 (17.7)	0.821
Antihypertensive drugs [*n* (%)]	41 (67.2)	34 (54.8)	0.197
Antidiabetic drugs [*n* (%)]	27 (44.3)	14 (22.6)	0.013
Anticoagulants [*n* (%)]	3 (4.9)	3 (4.8)	>0.999
Antiplatelet drugs [*n* (%)]	8 (13.1)	6 (9.7)	0.583
Lipid metabolism-related laboratory parameters			
small dense low-density lipoprotein cholesterol (mmol/L)	1.12 ± 0.43	1.10 ± 0.46	0.821
Apolipoprotein B (g/L)	0.91 ± 0.23	0.87 ± 0.25	0.372
Apolipoprotein E (mg/dL)	3.94 ± 1.33	4.64 ± 1.63	0.011
Apolipoprotein A1 (g/L)	1.18 ± 0.15	1.16 ± 0.17	0.523
Homocysteine (μmol/L)	14.66 ± 5.75	20.57 ± 17.28	0.013
Folate (ng/mL)	25.27 ± 14.42	22.41 ± 13.76	0.262
Vitamin B12 (pmol/L)	274.33 ± 117.87	314.18 ± 213.02	0.201
Glycated hemoglobin (%)	7.00 ± 1.60	6.75 ± 1.61	0.380
Glucose (mmol/L)	6.02 ± 2.52	5.69 ± 2.72	0.488

PSCI, post-stroke cognitive impairment; NIHSS, National Institutes of Health Stroke Scale; GCS, Glasgow Coma Scale; T-MoCA, telephone version of Montreal Cognitive Assessment Scale. The measurement data is expressed as mean ± standard deviation, and the counting data is expressed as an example (%); Post-stroke cognitive impairment was defined as a 3-month T-MoCA score < 19.

### 3.2 Quality control and overall characteristics of serum lipidomics

In this study, untargeted lipidomics using data-dependent acquisition (DDA) LC–MS/MS was performed on acute-phase serum samples collected within 24 h of stroke onset. After peak detection, lipid annotation, missing-value imputation, normalization, quality-control filtering, and batch-effect correction, a total of 1,584 lipid annotations meeting MSI Level 2 criteria were retained for subsequent analyses. Quality-control assessment indicated satisfactory analytical reproducibility of the lipidomics data (Supplementary Figure 1). The pooled QC samples clustered tightly in unsupervised PCA, suggesting stable instrument performance during data acquisition. After normalization and batch correction, the distribution of total lipid signal intensity was largely comparable across study samples. The RSD distribution of QC samples was predominantly concentrated in the low range, and the vast majority of lipid features had QC RSD values below the commonly used quality-control threshold of 30%, indicating good technical reproducibility of the retained lipid features. No obvious batch-related separation trend was observed after batch correction.

**Figure 2:**
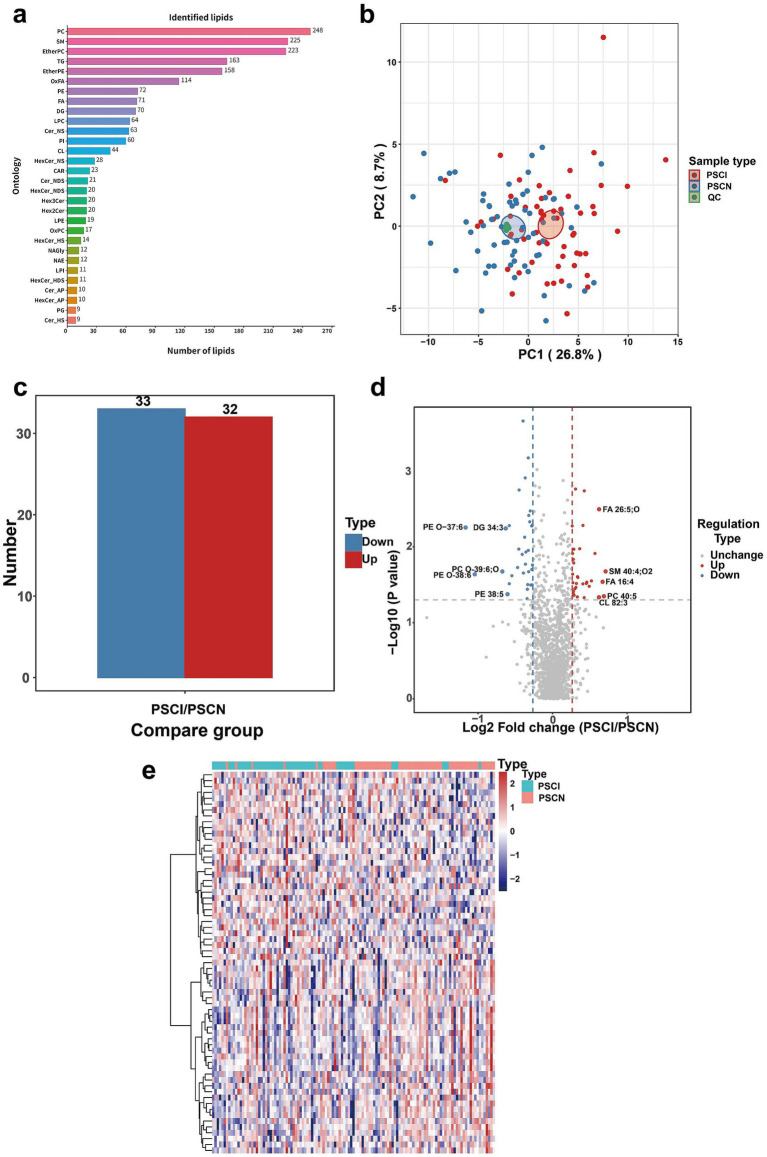
Serum lipidomic characteristics and exploratory candidate lipid identification between the PSCI and PSCN groups **(a)** Lipid-class distribution of the retained serum lipid annotations **(b)** Principal component analysis (PCA) score plot based on all retained lipid annotations, showing QC sample clustering and the distribution of PSCI and PSCN samples **(c)** Numbers of increased and decreased candidate lipid annotations after adjustment for clinical confounders **(d)** Volcano plot of candidate lipid annotations comparing the PSCI and PSCN groups **(e)** Hierarchical clustering heatmap of candidate lipid annotations. PSCI, post-stroke cognitive impairment; PSCN, post-stroke cognitively normal; QC, quality control.

The detected lipid annotations covered the major lipid subclasses present in serum, including glycerophospholipids, sphingolipids, neutral glycerolipids, ether lipids, and oxidized fatty acids. Among these, phosphatidylcholine (PC), sphingomyelin (SM), ether-linked phosphatidylcholine (EtherPC), triacylglycerol (TG), and ether-linked phosphatidylethanolamine (EtherPE) were the most abundant lipid classes, indicating that the lipidomics dataset provided broad coverage of the major components of the serum lipidome (Figure 2a).

### 3.3 Exploratory multivariate analysis and annotation of PSCI-associated differential lipids

Unsupervised PCA based on all retained lipid annotations showed tight clustering of QC samples, whereas PSCI and PSCN samples exhibited a modest separation trend along PC1 (PC1 = 26.8%, PC2 = 8.7%; Figure 2b). These findings suggest satisfactory analytical stability of the dataset and indicate that lipidomic differences may exist between the two groups. PLS-DA was used solely as an exploratory visualization method. The PLS-DA score plot showed a modest separation trend between the PSCI and PSCN groups (Supplementary Figure 2a). Supplementary Table 1 lists the lipid annotations ranked by VIP score. Cross-validation and 200 permutation tests indicated limited model robustness, with model parameters of R^2^X = 0.265, R^2^Y = 0.230, Q^2^ = 0.230, and a Q^2^ intercept of 0.042 (Supplementary Figure 2b). Therefore, PLS-DA was not used for candidate biomarker selection in this study.

In the univariate analysis, with an unadjusted *p* value < 0.05 as the threshold, 87 lipid annotations showed nominally significant differences between the PSCI and PSCN groups; of these, 34 were increased and 53 were decreased in the PSCI group. After Benjamini–Hochberg correction for the false discovery rate (FDR), no lipid remained statistically significant at FDR < 0.05. Therefore, lipid features identified using the unadjusted *p* < 0.05 threshold were regarded as exploratory candidate lipids only, rather than confirmed biomarkers.

To evaluate whether associations between these candidate lipids and PSCI were independent of clinical confounders, multivariable logistic regression was performed, with adjustment for diabetes mellitus, glucose-lowering medication use, apolipoprotein E, and homocysteine. After adjustment, 65 lipid annotations remained nominally associated with PSCI, including 32 increased and 33 decreased lipid annotations in the PSCI group (Figure 2c; Supplementary Table 2). Because these findings have not been externally validated, they should be interpreted as exploratory associations rather than definitive biomarkers.

The volcano plot showed that the confounder-adjusted candidate lipids were distributed across multiple lipid classes, including phosphatidylcholine (PC), phosphatidylethanolamine (PE), sphingomyelin (SM), fatty acids (FA), diacylglycerols (DG), and cardiolipins (CL) (Figure 2d). Hierarchical clustering heatmap analysis showed that these candidate lipids exhibited group-related abundance patterns between the PSCI and PSCN groups, although substantial overlap remained (Figure 2e). At the lipid-class level, oxidized fatty acids (OxFA), SM, lysophosphatidylinositol (LPI), CL, FA, and phosphatidylinositol (PI) showed an overall increasing trend in the PSCI group, whereas ether-linked phosphatidylcholine (EtherPC), PE, DG, ether-linked phosphatidylethanolamine (EtherPE), triacylglycerols (TG), cholesteryl esters (CE), and lysophosphatidylcholine (LPC) showed an overall decreasing trend in the PSCI group (Supplementary Figure 2). These results suggest that PSCI is associated with broad but relatively modest changes in the serum lipid profile, particularly involving lipid metabolic categories related to oxidized lipids, sphingolipids, glycerophospholipids, and neutral lipids.

### 3.4 Pathway enrichment analysis

**Figure 3:**
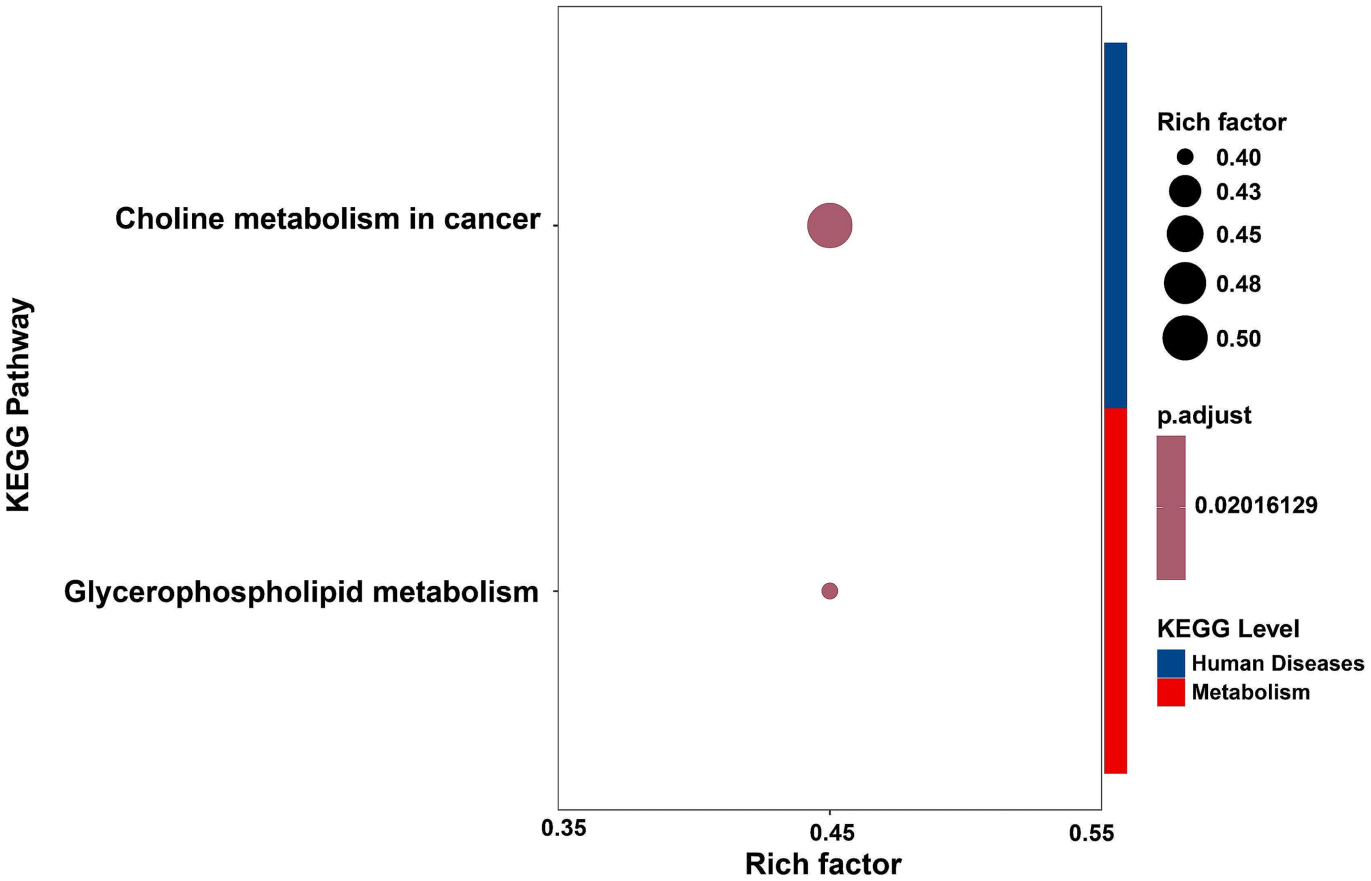
KEGG pathway enrichment analysis of candidate lipid annotations after adjustment for confounders. Bubble size indicates the number of enriched lipid annotations, the *x*-axis indicates the enrichment factor, and color represents enrichment significance.

The 65 candidate lipid annotations that remained nominally associated with PSCI after adjustment for confounders were used as the input set, whereas all lipid annotations retained after quality-control filtering were used as the background set for Kyoto Encyclopedia of Genes and Genomes (KEGG) pathway enrichment analysis. The top-ranked enriched KEGG pathways included “choline metabolism in cancer” and “glycerophospholipid metabolism” (Figure 3). The enrichment factor was approximately 0.50 for “choline metabolism in cancer” and approximately 0.40–0.48 for “glycerophospholipid metabolism.” It should be noted that “choline metabolism in cancer” is a KEGG database pathway name and does not indicate the presence of cancer-related biological processes in this stroke cohort. Rather, this result should be interpreted as enrichment of metabolic processes involving choline-containing phospholipids. Overall, the pathway enrichment results support an exploratory association between PSCI and alterations in glycerophospholipid and choline-related lipid metabolism; however, they do not allow causal or mechanistic inference.

### 3.5 Construction and internal validation of prediction models

**Figure 4:**
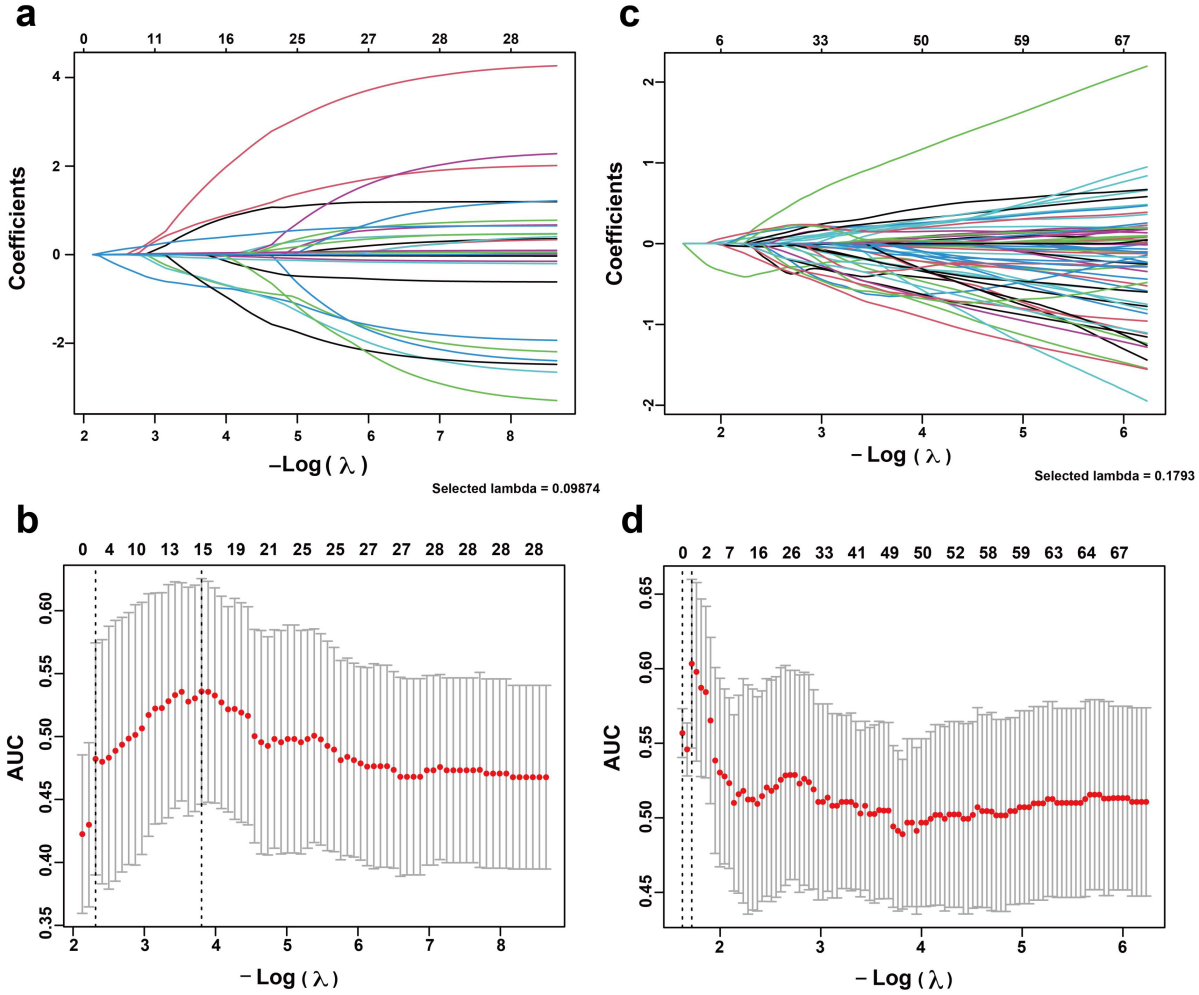
Selection of clinical and lipidomic variables using LASSO regression. **(a,b)** LASSO coefficient path plot and cross-validation curve for the clinical variables. **(c,d)** LASSO coefficient path plot and cross-validation curve for the lipidomic variables. The *x*-axis represents −log(*λ*), and the numbers at the top indicate the number of variables with nonzero coefficients at the corresponding *λ* value. Red dots indicate cross-validated area under the curve (AUC) values, gray error bars indicate standard errors, and dashed lines indicate the selected *λ* values.

The study cohort was randomly divided into a training set (*n* = 86) and an internal validation set (*n* = 37) at a 7:3 ratio. Baseline characteristics were comparable between the training and validation sets, with all *p* values > 0.05. All feature-selection and model-fitting procedures were performed exclusively in the training set. LASSO logistic regression with 10-fold cross-validation was used for feature selection. In the clinical-variable selection model, diabetes mellitus, apolipoprotein E, homocysteine, and glucose-lowering medication use were retained as variables with nonzero coefficients (Figures 4a,b). In the lipidomics-variable selection model, LPC 22:6/0:0 was retained as the selected lipidomic feature (Figures 4c,d). Subsequently, the clinical, lipidomic, and combined models were developed using the selected variables. In the training-set combined model, LPC 22:6/0:0 was associated with PSCI after adjustment for the selected clinical predictors, with an adjusted OR of 0.128 (95% CI, 0.036–0.453; *p* = 0.001), corresponding to a Cohen’s d of −1.134.

**Figure 5:**
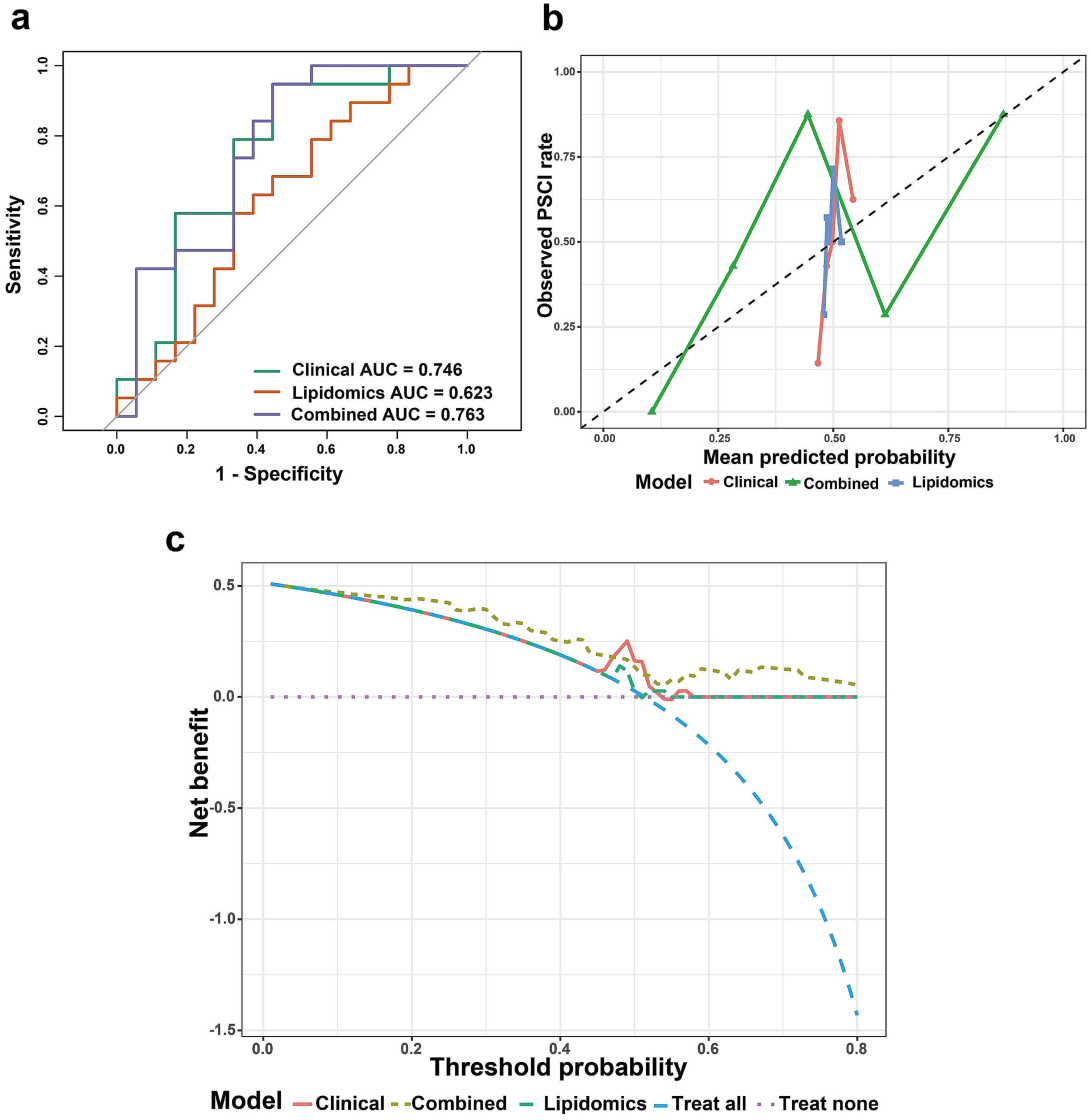
Predictive performance, calibration, and clinical utility of the clinical, lipidomic, and combined models in the internal validation set. **(a)** Receiver operating characteristic (ROC) curves of the three models. **(b)** Calibration curve showing the agreement between the predicted probability of PSCI and the observed occurrence of PSCI; the dashed line represents ideal calibration. **(c)** Decision curve analysis showing the net benefit of each model across a range of threshold probabilities. AUC, area under the curve; ROC, receiver operating characteristic.

In the training set, the AUCs of the clinical, lipidomic, and combined models were 0.738 (95% CI, 0.630–0.846), 0.741 (95% CI, 0.635–0.848), and 0.833 (95% CI, 0.747–0.920), respectively. In the internal validation set, the AUCs of the clinical, lipidomic, and combined models were 0.746 (95% CI, 0.577–0.914), 0.623 (95% CI, 0.436–0.810), and 0.763 (95% CI, 0.601–0.925), respectively (Figure 5a). The combined model had a sensitivity of 0.947, a specificity of 0.556, and a Brier score of 0.201. The calibration curve showed generally acceptable agreement between the predicted probability of PSCI and the observed occurrence of PSCI (Figure 5b).

Bootstrap resampling with 1,000 iterations was performed to assess optimism in internal validation. The mean optimism values of the clinical and lipidomic models were 0.147 and 0.153, respectively, with optimism-corrected AUCs of 0.591 and 0.652. The combined model had a mean optimism of 0.029 and an optimism-corrected AUC of 0.805 (Supplementary Table 3, Supplementary Figure 3). These results suggest that the combined model showed relatively stable internal performance in this dataset, although external validation in an independent cohort is required before clinical application.

DeLong testing showed that the difference in AUC between the combined and clinical models was not statistically significant (*p* = 0.814), whereas the difference between the combined and lipidomic models approached statistical significance (*p* = 0.084). Decision curve analysis showed that, across a threshold probability range of approximately 0.20–0.60, the combined model provided greater net benefit than the clinical model, lipidomic model, treat-all strategy, and treat-none strategy (Figure 5c). These findings suggest that the combined model may have potential utility for risk stratification; however, the results remain exploratory and require further validation in independent cohorts.

## 4 Discussion

In this prospective cohort study, we systematically analyzed acute-phase serum lipidomic profiles after ischemic stroke and their association with cognitive status 3 months after stroke. LPC 22:6/0:0, a docosahexaenoic acid–containing lysophospholipid, was selected as a candidate lipidomic feature by the LASSO model, suggesting that DHA-related lysophospholipid metabolism may be involved in post-stroke cognitive outcomes. By integrating clinical variables with the selected lipidomic feature, we developed an early risk assessment model with favorable performance. In the internal validation set, the combined model demonstrated favorable discrimination and calibration, as well as potential clinical net benefit.

First, our findings indicate that PSCI-related lipid alterations are characterized by broad, multi-class, and relatively modest metabolic remodeling, rather than pronounced changes in a single lipid class. This may be explained, at least in part, by the fact that both comparison groups consisted of patients with acute ischemic stroke. Patients in both groups were exposed to shared pathophysiological influences, including cerebral ischemia, inflammatory responses, oxidative stress, and vascular risk factors; therefore, between-group lipidomic differences may be more subtle than those observed between patients and healthy controls. Previous studies have suggested that post-stroke dysregulation of lipid metabolism may involve oxidative stress, impaired energy metabolism, altered sphingolipid signaling, and membrane phospholipid remodeling (31–34). In the present study, the candidate lipids spanned multiple lipid classes, including PC, PE, PI, LPC, SM, DG, and ether phospholipids, which is generally consistent with previous evidence indicating disruption of membrane lipid homeostasis and aberrant lipid signaling. In addition, previous clinical studies have reported that an elevated triglyceride-to-high-density lipoprotein cholesterol (TG/HDL-C) ratio and triglyceride-glucose (TyG) index are associated with an increased risk of PSCI, suggesting that peripheral lipid metabolic disturbances and insulin resistance–related metabolic states may contribute to post-stroke cognitive decline (35, 36). Animal studies have also shown that long-term cognitive impairment after stroke may be accompanied by phospholipid metabolic abnormalities, such as reduced PC levels and increased LPE and PS levels (37). Collectively, these findings support a potential link between dysregulated lipid metabolism and PSCI, although the present results require validation in independent cohorts.

Second, LPC 22:6/0:0, which was selected by LASSO regression, warrants particular attention. LPC 22:6/0:0 is a docosahexaenoic acid (DHA)-containing lysophospholipid. DHA is an important polyunsaturated fatty acid component of neuronal membranes and synaptic structures and is closely involved in membrane fluidity, synaptic plasticity, and inflammatory regulation (38–40). Previous studies have shown that LPC-bound DHA represents an important form of brain DHA uptake and can be transported across the blood–brain barrier via major facilitator superfamily domain-containing protein 2a (Mfsd2a) (41). Compared with free DHA, LPC-DHA may be more effective in increasing brain DHA levels and improving cognition-related functions (42). Therefore, the association between LPC 22:6/0:0 and PSCI has biological plausibility and may reflect differences in DHA-related lysophospholipid metabolism and peripheral metabolic signals associated with membrane lipid homeostasis. However, this study measured lipid profiles in peripheral serum, which may not directly reflect brain DHA levels or lipid metabolic status in the central nervous system. Thus, LPC 22:6/0:0 should not be interpreted as having definitive neuroprotective effects; rather, it should be regarded as a candidate lipid biomarker requiring further validation. The direction of changes in LPC-DHA–related molecules has not been fully consistent across studies, which may be attributable to differences in species, experimental models, sampling time windows, and detection platforms (34, 37).

In addition, we observed a decreasing trend in the abundance of several ether phospholipid–related lipids in the PSCI group. Ether phospholipids, especially plasmalogens, are important components of cell membranes and may play roles in antioxidant defense, anti-inflammatory responses, and the maintenance of membrane structural stability (43–46). Previous studies have reported that lipid alterations after stroke are time-dependent. The acute phase may be characterized by enhanced sphingolipid and glycosphingolipid signaling, whereas the subacute or chronic phase may involve more pronounced membrane lipid remodeling and inflammation-related lipid changes (37, 47). Feng et al. also suggested that ether phospholipids may participate in neuroprotective processes through anti-inflammatory and antioxidant mechanisms and by regulating membrane homeostasis (48). Taken together with our findings, reduced circulating ether phospholipid levels in the acute phase may be associated with antioxidant defense, inflammatory regulation, or membrane homeostasis–related processes. However, their specific functional implications require further validation using oxidative stress markers, inflammatory indicators, and imaging features of brain injury.

Pathway enrichment analysis suggested that PSCI may be associated with alterations in glycerophospholipid metabolism and choline-related lipid metabolism. Based on the 65 candidate lipid annotations that remained nominally associated with PSCI after adjustment for confounders, KEGG analysis showed enrichment of “glycerophospholipid metabolism” and “choline metabolism in cancer.” The latter is a standard pathway name in the KEGG database and does not indicate the presence of cancer-related biological processes in this stroke cohort. Rather, it more likely reflects alterations in metabolic processes involving choline-containing phospholipids. Choline-related phospholipid metabolism is involved in cellular membrane renewal, neurotransmitter synthesis, membrane-associated signal transduction, and inflammatory regulation. These pathway-level findings suggest that alterations in choline-containing phospholipid metabolism may represent an important component of lipid metabolic remodeling associated with PSCI. Previous studies have also suggested that lipotoxic accumulation, blood–brain barrier injury, and chronic inflammatory activation may contribute to cognitive deterioration (49, 50). However, because the lipids used for pathway analysis did not reach statistical significance after FDR correction, these results should be interpreted as exploratory.

Regarding prediction model development, this study showed that the combined clinical and lipidomic model outperformed the lipidomic model alone, with an AUC of 0.763. In the internal validation set, the combined model demonstrated favorable discrimination and calibration, as well as potential clinical net benefit. Previous studies have also suggested that models integrating multiple indicators perform better than single-marker models (51, 52), further supporting the potential value of multi-indicator or multi-omics integration strategies for PSCI prediction. However, the DeLong test showed that the increase in AUC for the combined model relative to the clinical model did not reach statistical significance, indicating that the incremental predictive value of the lipidomic feature remains limited. Therefore, this model should be regarded as an exploratory risk-stratification tool and should not yet be directly applied to clinical decision-making.

This study has several limitations. First, this was a single-center exploratory study with a relatively limited sample size and no independent external validation cohort, which limits the generalizability of the findings. Future multicenter studies with larger sample sizes, together with mechanistic experiments, are needed to further validate the functional roles of key lipid molecules. Second, serum lipidomics reflects peripheral systemic metabolic status and may not fully represent local lipid metabolic changes in the brain. Third, cognitive outcomes were assessed using the T-MoCA at 3 months, which improved the feasibility of follow-up but does not replace comprehensive face-to-face neuropsychological assessment or capture longer-term trajectories of cognitive change. Finally, Although MS-DIAL integrates accurate mass, isotopic patterns, MS/MS spectral matching, and lipid-class-specific fragmentation rules for lipid annotation, ambiguities may still arise from in-source fragmentation, co-elution, and isobaric or isomeric lipids, especially double-bond and sn-positional isomers that cannot be fully resolved without authentic standards or targeted validation. Therefore, the key candidate lipids require further confirmation using authentic standards and targeted quantitative methods.

In conclusion, this study provides preliminary lipidomic evidence of early serum lipid metabolic alterations in patients with PSCI after acute ischemic stroke. LPC 22:6/0:0, identified as a candidate lipidomic feature by the model, may reflect changes related to docosahexaenoic acid (DHA)-associated lipid transport and neuronal membrane homeostasis, although its biological function requires further validation. The model combining clinical variables and lipidomic features showed potential for risk stratification; however, confirmation in larger multicenter longitudinal studies is needed. Future studies should integrate targeted lipidomics, brain imaging phenotypes, and mechanistic experiments to further clarify the role of lipid metabolism in PSCI.

## 5 Conclusion

Based on a prospective cohort and untargeted serum lipidomic analysis, this study found that patients who developed PSCI after acute ischemic stroke exhibited early lipid metabolic remodeling characterized by broad, multi-class, and low-magnitude alterations. LPC 22:6/0:0, a DHA-associated lysophospholipid selected by the LASSO model, may be associated with cognitive outcomes after stroke. The prediction model integrating clinical variables and lipidomic features showed a certain degree of discrimination, calibration, and clinical net benefit in the internal validation set, providing exploratory lipidomic evidence for early risk stratification of PSCI.

## Data Availability

The raw data supporting the conclusions of this article will be made available by the authors, without undue reservation.
