# Supplementary material for: Serum lipidomic alterations associated with post-stroke cognitive impairment: an exploratory prospective study
Source: Front Neurol. 2026 Jul 29;17:1882198. doi: 10.3389/fneur.2026.1882198 (PMC13461449; doi:10.3389/fneur.2026.1882198)
Supplement: Supplementary file 1 [file Table_1.DOCX]

Supplementary Figure 1. Quality-control assessment of the serum lipidomics data. a, Partial least squares discriminant analysis (PLS-DA) score plot showing the distribution of PSCI, PSCN, and QC samples along the first two components, which explained 12.1% and 6.6% of the variance, respectively. Each point represents one sample, and ellipses indicate the distribution of different sample types. b, Boxplots showing the log2-transformed intensity distributions of detected lipid features across all samples. The comparable intensity distributions among samples indicate good signal stability.c, Distribution of the relative standard deviation (RSD) of detected lipid features in PSCI, PSCN, and QC samples. QC samples showed markedly lower RSD values than biological samples, supporting satisfactory analytical reproducibility and data quality. PSCI, PSCN, and QC samples are shown in red, blue, and green, respectively.

Supplementary Figure 2. PLS-DA analysis and lipid class-level alterations in serum lipidomic profiles. a, Partial least squares discriminant analysis (PLS-DA) score plot of serum lipidomic profiles from PSCI and PSCN samples, with QC samples included to assess analytical stability. The first two components, t1 and t2, explained 26.5% and 4.22% of the variance, respectively. Each point represents one sample, and shaded ellipses indicate the distribution of different sample types. b, Trend analysis of differential lipid classes between PSCI and PSCN samples. The bar plot shows the log2-transformed fold change of each lipid class in the PSCI/PSCN comparison. Positive values indicate lipid classes increased in PSCI relative to PSCN, whereas negative values indicate lipid classes decreased in PSCI relative to PSCN.

Supplementary Figure 3. Bootstrap internal validation of the prediction models. Bar plot showing the bootstrap internal validation results for the clinical, lipidomics, and combined models. Apparent area under the receiver operating characteristic curve (AUC), mean optimism, and optimism-corrected AUC are shown for each model. Numeric values are displayed above the corresponding bars.
