# Supplementary material for: Serum lipidomic alterations associated with post-stroke cognitive impairment: an exploratory prospective study
Source: Front Neurol. 2026 Jul 29;17:1882198. doi: 10.3389/fneur.2026.1882198 (PMC13461449; doi:10.3389/fneur.2026.1882198)
Supplement: Supplementary file 2 [file Table_2.DOCX]

| Lipid name | vip |
| --- | --- |
| PI 38:2\|PI 20:0_18:2 | 2.51571787636765 |
| ST 29:2;O;S | 1.64681421721047 |
| PC 40:5\|PC 16:0_24:5 | 1.43974736501013 |
| HexCer 42:3;O3\|HexCer 18:1;O2/24:2;O | 1.40084690104414 |
| TG 58:10\|TG 18:2_20:4_20:4 | 1.39687190705221 |
| TG 53:3\|TG 17:1_18:1_18:1 | 1.39293766251675 |
| TG 60:12\|TG 16:0_22:6_22:6 | 1.29516088590172 |
| LPC 22:6/0:0 | 1.2531126307352 |
| TG 55:4\|TG 18:1_19:1_18:2 | 1.23377645703915 |
| PC O-46:5\|PC O-24:0_22:5 | 1.21186808929968 |
| TG 53:3\|TG 17:0_18:1_18:2 | 1.20772857822666 |
| TG 58:11\|TG 18:2_18:3_22:6 | 1.16615941842271 |
| PC 42:10\|PC 20:4_22:6 | 1.15841217762091 |
| TG 54:8\|TG 14:0_18:2_22:6 | 1.15087870571927 |
| PC 39:6 | 1.15036753506829 |
| LPC 18:2/0:0 | 1.11670891603738 |
| TG 55:8\|TG 15:0_18:2_22:6 | 1.11100875836519 |
| TG 51:6\|TG 15:2_18:2_18:2 | 1.09238798799121 |
| TG 53:5\|TG 17:1_18:2_18:2 | 1.05875074731713 |
| PC 39:6\|PC 17:0_22:6 | 1.03402923074278 |
| PC O-32:2\|PC O-16:1_16:1 | 1.02654862338868 |
| TG 56:8\|TG 16:0_18:2_22:6 | 1.01800091566109 |
| PC 37:6\|PC 15:0_22:6 | 1.00724525216429 |
| TG 49:3\|TG 15:0_16:1_18:2 | 1.00673602323785 |
| FA 26:5;O | 0.958889288964163 |
| CL 70:1\|CL 16:0_16:0_20:0_18:1 | 0.958440759746333 |
| FA 16:4 | 0.956010321039884 |
| TG 55:6\|TG 17:0_18:2_20:4 | 0.949275270224511 |
| LPI 18:3 | 0.941342823826033 |
| PC 46:4\|PC 24:0_22:4 | 0.9295098523485 |
| SM 40:4;O2 | 0.914130316931521 |
| Hex3Cer 40:2;O2 | 0.910110545799657 |
| DG 42:7 | 0.905194044810018 |
| CE 22:6 | 0.902353241934751 |
| TG 55:7\|TG 17:1_18:2_20:4 | 0.892351088068807 |
| PC O-40:7\|PC O-18:2_22:5 | 0.889588026590748 |
| PC 37:5 | 0.874885302281128 |
| HexCer 41:0;O2 | 0.865923545874471 |
| PC 44:4\|PC 24:1_20:3 | 0.858386795679355 |
| TG 58:9\|TG 18:1_18:2_22:6 | 0.845298749357655 |
| PC 44:12\|PC 22:6_22:6 | 0.841534898117837 |
| TG 56:10\|TG 18:2_18:3_20:5 | 0.823914850939709 |
| FA 17:3 | 0.819806415938536 |
| PE O-38:6\|PE O-16:1_22:5 | 0.802287079411106 |
| DG 34:3 | 0.78894906138119 |
| PE O-37:6\|PE O-17:1_20:5 | 0.766610639451017 |
| PC 42:5\|PC 20:0_22:5 | 0.750992789626769 |
| PE 38:5\|PE 18:0_20:5 | 0.734107402062147 |
| SM 36:1;O2\|SM 19:0;O2/17:1 | 0.729504952669296 |
| PC O-39:6;O\|PC O-22:5_17:1;O | 0.723748708108496 |
| PE O-38:6\|PE O-18:1_20:5 | 0.720262797326885 |
| TG 52:5\|TG 16:1_18:2_18:2 | 0.716360442193879 |
| PC O-38:5\|PC O-16:1_22:4 | 0.708104332399355 |
| FA 24:1 | 0.68318565157839 |
| PC 40:8\|PC 18:2_22:6 | 0.676263361681817 |
| PC 42:4\|PC 22:1_20:3 | 0.635209561691091 |
| PC O-46:8\|PC O-24:3_22:5 | 0.629617797430041 |
| PE O-40:2\|PE O-18:1_22:1 | 0.620684340614662 |
| PC O-36:1\|PC O-20:1_16:0 | 0.613391526429098 |
| HexCer 39:2;O2 | 0.590378027292275 |
| ASG 27:1;O;Hex;FA 18:0 | 0.571350666705467 |
| CL 82:3\|CL 18:0_18:0_26:0_20:3 | 0.540113297220712 |
| LPC 24:5 | 0.531623308810427 |
| Hex2Cer 34:0;O2 | 0.466203625230967 |
| Hex3Cer 44:2;O2 | 0.435959867970002 |

Supplementary Table 1. VIP, Variable Importance in Projection; PLS-DA, partial least squares discriminant analysis. Lipids are listed in descending order of VIP value.

| Rank | Lipid name | ratio | -log_10_(P) | Direction of change |
| --- | --- | --- | --- | --- |
| 1 | PC 37:6\|PC 15:0_22:6 | 0.75973 | 3.6548 | Down |
| 2 | PC 39:6 | 0.797833 | 3.1701 | Down |
| 3 | LPC 22:6/0:0 | 0.775557 | 2.9061 | Down |
| 4 | FA 24:1 | 1.239157 | 2.7596 | Up |
| 5 | PC 39:6\|PC 17:0_22:6 | 0.732796 | 2.7465 | Down |
| 6 | PC 46:4\|PC 24:0_22:4 | 1.343335 | 2.7360 | Up |
| 7 | FA 26:5;O | 1.541681 | 2.4938 | Up |
| 8 | TG 53:3\|TG 17:1_18:1_18:1 | 0.812783 | 2.4719 | Down |
| 9 | TG 52:5\|TG 16:1_18:2_18:2 | 0.801825 | 2.4135 | Down |
| 10 | PC 42:10\|PC 20:4_22:6 | 0.809084 | 2.3293 | Down |

Supplementary Table 2. Top 10 exploratory differential lipid species between PSCI and PSCN.

The table lists the top 10 exploratory differential lipid species based on unadjusted between-group comparisons. The ratio represents lipid abundance in PSCI relative to PSCN; ratio >1 indicates increased abundance in PSCI, and ratio <1 indicates decreased abundance. −log10(P) represents the negative log10-transformed unadjusted P value. PSCI, post-stroke cognitive impairment; PSCN, post-stroke cognitively normal.

| Model | apparent_auc | mean_optimism | optimism_corrected_auc | B_total | B_success |
| --- | --- | --- | --- | --- | --- |
| Clinical | 0.738236884802596 | 0.146939957830545 | 0.591296926972051 | 1000 | 1000 |
| Lipidomics | 0.805300162249865 | 0.153132126625943 | 0.652168035623921 | 1000 | 1000 |
| Combined | 0.833423472147107 | 0.0285031287524978 | 0.804920343394609 | 1000 | 1000 |

Supplementary Table 3. Bootstrap internal validation of the clinical, lipidomics, and combined prediction models. The table summarizes the bootstrap internal validation results for the three prediction models. Apparent AUC represents the model performance estimated in the original dataset. Mean optimism represents the average difference between bootstrap-derived and test performance across resampling iterations. Optimism-corrected AUC was calculated by subtracting mean optimism from the apparent AUC. B_total indicates the total number of bootstrap resamples, and B_success indicates the number of successfully completed bootstrap iterations. AUC, area under the receiver operating characteristic curve.
